# Interpretation of evidence in data by untrained medical students: a scenario-based study

**DOI:** 10.1186/1471-2288-10-78

**Published:** 2010-08-26

**Authors:** Thomas V Perneger, Delphine S Courvoisier

**Affiliations:** 1 Division of Clinical Epidemiology, Geneva University Hospitals, Geneva, Switzerland and Faculty of Medicine, University of Geneva, Geneva, Switzerland

## Abstract

**Background:**

To determine which approach to assessment of evidence in data - statistical tests or likelihood ratios - comes closest to the interpretation of evidence by untrained medical students.

**Methods:**

Empirical study of medical students (N = 842), untrained in statistical inference or in the interpretation of diagnostic tests. They were asked to interpret a hypothetical diagnostic test, presented in four versions that differed in the distributions of test scores in diseased and non-diseased populations. Each student received only one version. The intuitive application of the statistical test approach would lead to rejecting the null hypothesis of no disease in version A, and to accepting the null in version B. Application of the likelihood ratio approach led to opposite conclusions - against the disease in A, and in favour of disease in B. Version C tested the importance of the p-value (A: 0.04 versus C: 0.08) and version D the importance of the likelihood ratio (C: 1/4 versus D: 1/8).

**Results:**

In version A, 7.5% concluded that the result was in favour of disease (compatible with p value), 43.6% ruled against the disease (compatible with likelihood ratio), and 48.9% were undecided. In version B, 69.0% were in favour of disease (compatible with likelihood ratio), 4.5% against (compatible with p value), and 26.5% undecided. Increasing the p value from 0.04 to 0.08 did not change the results. The change in the likelihood ratio from 1/4 to 1/8 increased the proportion of non-committed responses.

**Conclusions:**

Most untrained medical students appear to interpret evidence from data in a manner that is compatible with the use of likelihood ratios.

## Background

Despite the advent of evidence-based medicine, assessing "evidence" in data is no easy task. Doctors are confronted with evidence in two situations: when interpreting the results of laboratory tests during patient care, and when interpreting statistical tests reported in scientific articles. These situations are similar in many ways. In both situations one wants to infer the true state of things (Does the patient have the disease or not? Is the scientific hypothesis true or false?) from an observed result (a positive or negative diagnostic test, a statistical test that is significant or not) [1].

Two approaches to statistical inference currently coexist: statistical tests and likelihood ratios. The statistical test approach considers the probability of the observed result, and of more extreme results, under the null hypothesis - i.e., the p value. If the p-value is large the observation is considered compatible with the null hypothesis. If the p-value is small, the test is deemed "significant" and the null hypothesis is rejected (we describe here a common conflation of the Neyman-Pearson test theory, which considers only the rejection or acceptance of the null hypothesis, and Fisher's use of the p value, which is seen as inversely related to the strength of evidence against the null hypothesis). The alternate hypothesis is a passive bystander in this analysis; it is selected by default when the null hypothesis is rejected.

In contrast, the likelihood ratio approach relies on the direct comparison of the probabilities (or probability densities) of the observed result under two hypotheses [2-6]. The hypothesis under which the result is the more likely is considered supported by the data, and the strength of support is given by the ratio of the probabilities (the likelihood ratio). The key difference with a statistical test is that *two *distributions must be compared.

Several interpretations of evidence also coexist in the realm of diagnostic tests. While p values are not used in this context, the traditional interpretation is based on the consideration of error probabilities under the hypotheses of presence of disease (1-sensitivity, type 2 error) and absence of disease (1-specificity, type 1 error). Also, many laboratory tests come with an interval of "normal values" that is conceptually similar to the acceptance region of a statistical test. The likelihood ratio is increasingly used in the interpretation of diagnostic tests [2].

What approach to inference is better is currently debated. P-values have come under fire [7-13], and the use of likelihood ratios has gained support, particularly among methodologists [5-7]. Neither method has been endorsed by the medical community; available evidence suggests that many doctors are unable to interpret p-values correctly [14] or to use likelihood ratios [15]. Missing from this debate is the examination of how people with no knowledge of statistical inference interpret the world around them. This is important for two reasons. Firstly, the inference method should conform to common sense, or else it will be misused or misinterpreted. Secondly, humans have evolved over millennia the basic tools for decision-making in a complex world, sometimes called heuristics [16], that are simple yet reliable in everyday situations - thus an intuitive approach to interpretation of data comes with a Darwinian seal of approval. Little is known about the intuitive assessment of evidence by untrained people.

This study examined which approach to the assessment of evidence in data - statistical tests or likelihood ratios - was more compatible with the interpretation of data by first year medical students, who were asked to draw inferences from a diagnostic scenario. Since these students have not been trained, either in statistics or in the interpretation of clinical data, their answers may capture the natural data interpretation strategy of the human mind.

## Methods

Three cohorts of first year medical students at the School of Medicine, University of Geneva, were invited to complete a questionnaire during the first class of the course "Statistics for doctors", in 2005, 2009 and 2010, before any teaching occurred. The questionnaire included several scenarios involving the interpretation of data. The diagnostic scenario analyzed here was produced in 4 versions: versions A and B were tested in 2005 and 2009, and versions C and D in 2010. The 2 versions of a given year were distributed in a haphazard way: the two types of questionnaires were shuffled, much like a deck of cards, and handed out in small packets across the auditorium. Students were not informed that there were 2 versions. They were asked to select answers that appeared the most suitable. The questionnaire was anonymous.

The diagnostic scenario described a patient with a suspected disease who is administered a diagnostic test (Table 1). The scenario was accompanied by a table of frequency distributions of diseased and non-diseased individuals across values of the test. This table differed for the four versions of the questionnaire. The respondent was asked whether the test result was an argument for the disease, against the disease, or neither for nor against. The latter group were also labelled "non-committed respondents". Respondents were also asked to rate the strength of evidence, from very strong to nil, on a five point scale.

**Table 1:** Diagnostic dilemma submitted to 1^st ^year medical students.

You are on call at the emergency room. A patient arrives, Mr. Fender, who aches all over. You suspect, among other diseases, an **acute ravepartitis**. You know that the Hendrix test can orient you as to the presence of this disease. This test consists in having the patient listen to "Star spangled banner" played by Jimi Hendrix at Woodstock in 1969, at 100 dB, and in counting the number of seconds until the patient screams and covers his/her ears. Large studies, based on thousands of observations, have shown that people who have and have not this disease are distributed as follows, according to the Hendrix test (only one version shown):								
	**Version A**		**Version B**		**Version C**		**Version D**	

**Hendrix test (seconds)**	**Ravepartitis absent (%)**	**Ravepartitis present (%)**	**Ravepartitis absent (%)**	**Ravepartitis present (%)**	**Ravepartitis absent (%)**	**Ravepartitis present (%)**	**Ravepartitis absent (%)**	**Ravepartitis present (%)**

0-10	0	87	0	15	1	87	0	87

11-20	0	11	3	40	3	11	0	11

**21-30**	**4**	**1**	**8**	**32**	**4**	**1**	**8**	**1**

31-60	21	1	36	11	21	1	36	1

61 or more	75	0	53	2	71	0	56	0

Total	100	100	100	100	100	100	100	100

You administer this test to Mr. Fender who starts to scream after **25 seconds**. Is this result an argument for or against the diagnosis of ravepartitis? For/Against/Neither for nor against

In version A, the patient's test value was reported for 4% of non-diseased individuals and 1% of diseased individuals. This corresponds to a likelihood ratio of 4 in favour of absence of the disease. The probability of the observed or more extreme results under the null hypothesis of no disease was 0.04, which would lead to rejection of this hypothesis at the usual threshold of <0.05. Thus if one applies the likelihood ratio approach, the test result argues against the presence of disease, and if one applies the statistical test approach, the test result leads to rejection of the null hypothesis of no disease, i.e., evidence for the presence of disease.

In version B, the test value was reported for 8% of non-diseased individuals, and 32% of diseased individuals. This too corresponds to a likelihood ratio of 4, but this time in favour of the presence of the disease. The probability of the observed or more extreme results under the null hypothesis of no disease was 11% (i.e., the sum of observed and more extreme probabilities: 0.08, 0.03 and 0.00, which corresponds to a one sided p = 0.11), a non-significant result. Students who applied the likelihood ratio approach would interpret the test result as evidence for the disease, whereas those who applied the statistical test approach would fail to reject the null hypothesis, evidence against the disease.

Version C was based on version A, but with the p-value increased to 0.08 (i.e., the sum of observed and more extreme probabilities: 0.04, 0.03 and 0.01). The purpose was to see if a non-significant result by usual criteria would increase the proportion of respondents who found that the test result favoured no disease. Version D was based on version C, but with the likelihood ratio strengthened to 1/8 (from 1/4). The purpose was to see if a steeper ratio would increase the proportion of respondents who found that the test result favoured no disease.

We cross-tabulated the responses by type of scenario, comparing proportions of opinions that were compatible with the likelihood ratio approach, the statistical test approach, and the non-committed answers. The distributions were compared using a chi-square test. We repeated these analyses after exclusion of the non-committed respondents. We compared versions A vs. B, A vs. C, and C vs. D. We performed the same comparisons for the ratings of strength of evidence, restricting this analysis to the respondents who gave the majority opinion.

## Results

In total, 847 students returned their questionnaire: 282 in version A, 246 in version B, 166 in version C, and 163 in version D. This corresponds to about 80% of the number of students enrolled. Among the respondents were 556 (65.7%) women and 290 men (34.3%, 1 missing), and 794 (94.2%) were 18-23 years old (4 missing). A majority (590, 69.7%) had taken an advanced mathematics and science option for their baccalaureate, at age 18 or 19. The amount of previous statistics courses was >10 hours for 234 (27.6%) students, 1-9 hours for 203 (24.0%), and none for 410 (48.4).

Versions A and B implied opposite interpretations of the test according to the underlying approach to inference, either statistical test or likelihood ratio. In version A the statistical test approach was in favour of the disease and the likelihood ratio approach against, and in version B the pattern was opposite. In both cases (Table 2), more respondents answered according to the likelihood ratio approach (A: 43.6%, B: 69.0%) than the statistical test approach (A: 7.5%, B: 4.5%). Among those with a stated opinion (either for or against disease), the proportion who responded in a way that was compatible with the likelihood ratio approach was somewhat lower for version A than version B (85.3% vs. 93.9%, p = 0.014). A fairly high proportion of respondents did not express an opinion about the meaning of the test result, and this proportion of non-committed respondents was higher for version A than B. Among respondents who gave the majority opinion (against disease in version A, for in version B), the distribution of strength of evidence was shifted to lower ratings for version A than for version B.

**Table 2:** Version characteristics, distributions of student responses, and comparisons of versions of the scenario.

	Version A	Version B	Version C	Version D	A vs. B	A vs C	C vs. D
P value (H_0_: absence of disease)	0.04	0.11	0.08	0.08			
			
Evidence from p value favours	Disease	No disease	No disease	No disease			
			
Likelihood ratio (LR) in favour of disease	0.25	4	0.25	0.125			
			
Evidence from LR favours	No disease	Disease	No disease	No disease			

Interpretation of result:					p < 0.001	p = 0.34	p = 0.020
For	21 (7.5)	169 (69.0)	9 (5.5)	5 (3.3)			
Against	122 (43.6)	11 (4.5)	83 (50.3)	56 (36.8)			
Neither for nor against	137 (48.9)	65 (26.5)	73 (44.2)	91 (59.9)			

Interpreted the result as for or against disease	143 (51.1)	180 (73.5)	92 (55.8)	61 (40.1)	p < 0.001	p = 0.34	p = 0.005

Interpretation excluding the undecided					p < 0.001	p = 0.27	p = 0.74
For	21 (14.7)	169 (93.9)	9 (9.8)	5 (8.2)			
Against	122 (85.3)	11 (6.1)	83 (90.2)	56 (91.8)			

Strength of evidence among majority opinion	(N = 122)	(N = 169)	(N = 83)	(N = 56)	P < 0.001 linear trend	p = 0.43 linear trend	p = 0.58 linear trend
Very strong	5 (4.1)	2 (1.2)	1 (1.2)	3 (5.4)			
Strong	22 (18.0)	54 (32.0)	13 (15.7)	12 (21.4)			
Moderate	39 (32.0)	95 (56.2)	28 (33.7)	12 (21.4)			
Weak	51 (41.8)	17 (10.1)	39 (47.0)	26 (46.4)			
Absent	5 (4.1)	1 (0.6)	2 (2.4)	3 (5.4)			

Version C differed from version A only in that the p-value changed from 0.04 to 0.08. Since the latter value would be considered non-significant by most people who apply statistical tests, fewer such respondents should rule in favour of disease under C than under A. In contrast, the likelihood ratio was identical in A and C. There were no statistically significant differences between scenario A and C (Table 2).

Version D differed from version C only in the likelihood ratio, while the p value was held constant. The only notable difference was that more respondents were uncommitted when the likelihood ratio was stronger, which ran against expectations. The proportions who ruled against the disease among those with a clear opinion were similar for C and D, and the ratings of strength of evidence were similar as well.

## Discussion

The majority of first year medical students appeared to interpret evidence provided by a clinical test by comparing probabilities in a way that is compatible with the likelihood ratio approach to inference. In contrast, only a minority gave answers that were consistent with the statistical test approach. This suggests that the comparison of likelihoods may be more intuitive and easier to understand and to apply than statistical testing. Others have argued that doctors use a form of Bayesian reasoning in establishing a diagnosis [17]. Our results may also explain why many doctors, and several noted statisticians, have difficulty accepting the logic behind statistical tests as measures of evidence.

Indeed, a key argument against statistical tests and p-values as measures of evidence has been their problematic logic. In the words of Harold Jeffreys [18]: *"I have always considered the arguments for the use of P values absurd. They amount to saying that a hypothesis that may or may not be true [...] has not predicted something that has not happened." *Other statisticians have noted that *"the argument seems to be basically illogical" *[8], have complained about the *"convoluted reasoning necessary to interpret a P-value" *[10], and have lamented *"why do we turn probability logic on its head in this way?" *[11]. These difficulties contrast with the simplicity of the likelihood ratio: if the observed result is more likely under hypothesis A than under hypothesis B, it argues in favour of A over B.

While most modern commentators find fault with statistical tests as measures of evidence, this criticism is not universal [19]. More importantly, statistical tests remain ubiquitous in published statistical analyses [13]. Either the arguments against tests are not compelling, or the medical research enterprise has such inertia that changing the way statistical analysis is conducted is very difficult.

Our results describe assessments of evidence by untrained respondents, they do not support or contradict any theory of inference. A scientific theory that contradicts intuitive reasoning is not necessarily incorrect. E.g., the theory of relativity leads to counter-intuitive statements about time and space yet is considered accurate. Furthermore, psychologists have described many examples where informal thought processes can be in error [20]. However, informal thought processes are correct most of the time when applied in familiar situations, and inferring how the world works from observations is a familiar task. Therefore the lack of endorsement by medical students of the statistical test approach to inference raises a red flag about this inference method.

Notably, students as a group did not apply the likelihood ratio approach by the book. Firstly, they did not interpret in the same way 2 situations characterized by the same likelihood ratio, if inversed (versions A and B). The evidence provided by probabilities of 32% versus 8% appeared to be more compelling than evidence based on 4% versus 1%. Possibly, the absolute difference between the probabilities influenced the interpretation of the data, and not merely their ratio. This is contrary to the law of likelihood, which states that the likelihood ratio captures all the information there is about the relative plausibility of the competing hypotheses. Secondly, version A (4% versus 1%) was characterized by a higher proportion of non-committed respondents than version B (32% versus 8%). It is possible that the students looked at the whole distributions of diseased and non-diseased individuals, noted that the observed result fell in-between the two modes, in a grey zone, and concluded that this was an indeterminate result. If so, they would have considered the probability of events that had not happened, in Jeffreys' words. It is even possible that some may have performed statistical tests of both hypotheses, and rejected both in version A. Finally, a likelihood ratio that was twice as large (1/8 versus 1/4 in scenarios D and C) was not interpreted as stronger evidence for the more likely hypothesis; disturbingly, the proportion of undecided responses was greater for the more contrasted likelihood ratio. For these reasons, we cannot take our results as an endorsement of the likelihood ratio as a measure of evidence in data. Others too have observed that students do not apply Bayes' theorem when interpreting test results [21].

A minority of students did respond as though they applied the statistical test approach to inference. Furthermore, it is in principle possible to test any hypothesis, not just the null. If some students tested the hypothesis of "presence of disease", they would have concluded similarly to those who applied the likelihood ratio approach. This issue illustrates a key limitation of this study, namely the lack of a direct description of the respondents' thought process in interpreting the data. We only assessed the end product of the respondents' reasoning, which may have been reached by a variety of considerations. Qualitative studies may be necessary to better understand how students interpret data.

An unexpected result was the high proportion of non-committed respondents, in all versions of the scenario. This may simply reflect a lack of familiarity with the task on the students' part. Alternatively, it may reflect the fact that the observed result was not very compelling, by either approach. P-values of 0.04 or 0.08 are uncomfortably close to the conventional significance limit of 0.05; a likelihood ratio of 4 or 1/4 can be considered as weak evidence [2,3]. But assessment problems may also have contributed to the high proportion of non-committed answers. E.g., it is possible that some respondents have not understood the problem statement, or that some may have found it expedient to answer the equivalent of "I don't know" instead of reflecting on the data at hand. The scenarios we have used had not been tested with regard to their reliability and validity. This casts some doubt on the results presented in this paper. However, we believe that the contrasts between the versions of the scenario are such that measurement error is not a likely explanation of the main findings.

Another limitation of this study is that we asked a very narrow question (Is this result an argument for or against the diagnosis of ravepartitis), which may be unrealistic. In real life, doctors integrate the pre-test probability of disease with the test result in order to arrive at a post-test probability. This can be done through the application of the Bayes theorem, which few doctors are capable of doing [15], or by using natural frequencies, an easier and less error-prone method [22]. Our results suggest that the idea of comparing distributions under competing hypotheses has intuitive appeal, but do not guarantee that the correct interpretation of test results is easy or intuitive.

We should also acknowledge that the application of the likelihood ratio approach in research may prove challenging in some situations. One issue is the choice of an appropriate pair of hypotheses. Others are the lack of adequate statistical software, and the inertia of generations of medical researchers trained to do statistical tests. To bring home these arguments, even though we find the likelihood ratio approach conceptually appealing, we obtained P-values for the comparisons of groups of respondents in this study.

## Conclusions

In aggregate, these results suggest that untrained students might interpret data through a comparison of likelihoods, possibly by considering likelihood ratios. Promoting the use of likelihood ratios to communicate clinical or scientific results may facilitate the interpretation of evidence by doctors.

## Competing interests

The authors declare that they have no competing interests.

## Authors' contributions

TP proposed the initial study, analysed the results, wrote the paper. DC participated in the extensions to the initial protocol and in the interpretation of the results, and revised the paper. Both authors read and approved the submitted manuscript.

## Authors' information

Both authors teach statistical methods and clinical research methods to medical students, and both act as methods experts on clinical research projects

Funding: none

## Pre-publication history

The pre-publication history for this paper can be accessed here:

http://www.biomedcentral.com/1471-2288/10/78/prepub
